# The plant strengthening root endophyte *Piriformospora indica*: potential application and the biology behind

**DOI:** 10.1007/s00253-012-4506-1

**Published:** 2012-10-31

**Authors:** P. Franken

**Affiliations:** 1 Leibniz Institute of Vegetable and Ornamental Crops, Theodor-Echtermeyer-Weg 1, 14979 Großbeeren, Germany; 2 Humboldt University, Institute for Biology, Berlin, Germany

**Keywords:** Abiotic stress tolerance, Induced resistance, Inoculum formulation, *Piriformospora indica*, Plant growth promotion, *Sebacinales*

## Abstract

The successful conversion of plant production systems from conventional resource-exhausting to sustainable strategies depends on knowledge-based management of environmental factors. Root-inhabiting fungi came more and more into focus because their hyphae connect in ideal manner resources and challenges of the surrounding with the plant. A paradigm for such root endophytes is presented by the basidiomycete *Piriformospora indica*. This fungus possesses a broad host spectrum and positively affects different aspects of plant performance. This so far unique combination of attributes makes *P. indica* and its close relatives among the *Sebacinales* very interesting tools for cultivation of various crops. This review will outline the different aspects required to apply this root endophyte in agri- and horticulture concerning plant growth, plant nutrition and plant defence or tolerance thereby explaining what is known about the biological basis for the observed effects. Open questions and challenges for successful inoculum production and application will be discussed.

## Introduction

The rhizosphere of natural and anthropogenic ecosystems is inhabited by a plethora of organisms in which fungi constitute a large part of the biomass. Many of these rhizosphere fungi are able to colonise the plant and form different types of mycorrhiza (Smith and Read 2008). In addition, it has become evident that all plants also harbour non-mycorrhizal root-endophytic fungi, and their colonisation often impacts plant growth and development (bioregulation), plant nutrition (biofertilisation) and plant tolerance and resistance to abiotic and biotic stresses (bioprotection). Therefore, root-endophytic fungi have to be taken into account in order to understand the interaction of the root with its environment, and moreover, they could be used as biological agents to improve plant production systems.

Root endophytes with application potential can be found among non-pathogenic isolates of pathogens (Paparu et al. 2006) and among mycoparasites (Chacon et al. 2007). A morphologically defined group, the dark septate endophytes, which have been isolated from numerous plants (Jumpponen and Trappe 1998), including crops (Andrade-Linares et al. 2011), are represented by different species among the *Ascomycota*. A phylogenetically defined group are the *Sebacinales* (*Basidiomycota*). They contain mycorrhiza-forming and non-mycorrhizal root colonisers and occur worldwide (Weiß et al. 2011). The best-studied member is the species *Piriformospora indica*. It was originally isolated from the spore of an arbuscular mycorrhizal fungus found in the Thar Desert in India and root-colonising abilities were shown (Verma et al. 1998; isolate DSM 11827 deposited at the Deutsche Sammlung für Mikroorganismen und Zellkulturen, Braunschweig, Germany). In addition of forming orchid mycorrhiza (Blechert et al. 1998), plant growth-promoting effects were revealed for various hosts, and its application to plant production was proposed (Varma et al. 1999). Such a potential was further substantiated by the finding that barley plants colonised by *P*. *indica* were more resistant to pathogens and more tolerant to salt stress and showed higher yield (Waller et al. 2005).

Basic research to investigate the mechanisms of the interaction between *P*. *indica* and plants was facilitated by the fact that the fungus interacts with *Arabidopsis thaliana* (Peskan-Berghofer et al. 2004). Using this model, a number of genes were identified as being involved in the interaction (for review: Oelmuller et al. 2009). Recent establishment of a transformation system and the full genome sequence of the fungus (Zuccaro et al. 2009, 2011) will likely stimulate great progress towards further functional analysis. The positive effects observed for all tested plant species implicated a biotrophic interaction between the fungus and its host. However, staining of roots with fluorescein diacetate revealed an increase in dead root cells after colonisation (Franken et al. 2000). This phenomenon is not due to necrotrophic features of the endophyte (Schäfer et al. 2009), but rather it is due to increased programmed cell death (Deshmukh et al. 2006) triggered by endoplasmatic reticulum stress and caspase 1-like activity (Qiang et al. 2012a). The current view is that the interaction starts with a short biotrophic phase followed by further saprophytic feeding on dead plant cells (Zuccaro et al. 2011). During the different stages of root colonisation, plant innate immunity is down-regulated by manipulating several different phytohormone signalling pathways in order to facilitate a compatible interaction between the endophyte and the plant (Schäfer et al. 2009; Jacobs et al. 2011).

Recently, several reviews have focused on the interaction of *P*. *indica* with plants (Schafer et al. 2007; Oelmuller et al. 2009; Qiang et al. 2012b; Lahrmann and Zuccaro 2012). While these reviews mention potential applications, they are more concentrated on the mechanisms of interaction between the root endophyte and some model plants. Here we review significant publications related to the presence or absence of fungal effects on plant growth, plant nutrition, defence and tolerance, as well as on yield and plant product quality. The mechanisms and processes behind these effects have been investigated and described. Finally, progress and bottlenecks for inoculum production will be delineated in order to discuss future experiments which should smooth the way towards the application of *P*. *indica*.

## Plant growth and development

The most obvious effect of *P*. *indica* on plants is the promotion of vegetative growth, and this has been repeatedly shown with species from various plant families (Table 1). The extent of growth promotion is typically around 50 %, but significant variation exists, likely due in part to a number of environmental and experimental conditions. Thus far, analysis has only been carried out on the influence of substrate and the timing of inoculation on growth promotion (Fakhro et al. 2010). Following the course of growth parameters indicated that *P*. *indica* promoted initial stages of plant development (Barazani et al. 2005; Rai and Varma 2005). Further on, promotion of initial stages of vegetative growth results in an earlier switch to generative stages (Barazani et al. 2005; Achatz et al. 2010; Andrade-Linares et al. 2012). Promotion of early growth stages seems to be mainly based on accelerated root development (Waller et al. 2005; Baltruschat et al. 2008), and age-dependent regulation of genes was shifted to earlier time points in *P*. *indica*-colonised roots (Waller et al. 2008). Promotion of root development is an interesting feature as such. Indeed, application of *P*. *indica* results in enhanced rooting of callus cultures (Varma et al. 1999) and cuttings in the production of medicinal and ornamental plants (Rai and Varma 2005; Drüge et al. 2007).

**Table 1:** Plant growth promotion by *Piriformospora indica* measured as increases in fresh weight (fw) or dry weight (dw). Date of harvest is given in days after inoculation (dai)

	Plant material	Inoculation	Cultivation (conditions)	Biomass ratios inoculated/control	Reference
Various species (e.g. maize, poplar, parsley)	Seedlings or plantlets	Mycelium and spores mixed with expanded clay	4-week pot cultures in greenhouses (25 °C; 16 h light)	1.8–2.8 (shoot fw)	Varma et al. (1999)
				1.5–3.5 (root fw)	
*Spilanthes calva*	Seeds	Seed coating + mix with field soil	13 weeks field-grown (Central India)	2.3 or 7.8 (shoot dw)	Rai et al. (2001)
*Withania somnifera*				1.4 or 3.3 (root dw)	
*Arabidopsis thaliana*	10-day-old seedlings	Agar plug	On MM medium, 10 % P and N, no carbohydrates, continuous illumination	1.4 (shoot fw, 8 dai)^a^	Peskan-Berghofer et al. (2004)
				1.4 (root fw, 5 dai)^a^	
*Adhatoda vasica*	Cuttings	Mycelium and spore suspension to cuttings in water	Pot cultures with soil/sand/farmyard manure (3:1:1); (22 °C; day light; Central India)	2 (whole plants after 2 months)	Rai and Varma (2005)
				1.2 (whole plants after 6 months)	
*Nicotiana attenuata*	Seeds	Germination on plates ± fungus	Pot cultures	1.9 (seed germination 4 dai)^a^	Barazani et al. (2005)
				1.2 (stalk length 40 dai)^a^	
*Nicotiana tabacum*	14-day-old seedlings	Agar plug	On MM medium, 10 % P and N, no carbohydrates, continuous illumination	1.4 (seedling fw and dw)	Sherameti et al. (2005)
*Hordeum vulgare*	Seeds	Mycelium and spores mixed with expanded clay	5-week pot cultures in green houses (22/18 °C; 16 h light)	1.7 (shoot fw)^a^	Waller et al. (2005)
*Triticum aestivum*	Seedlings	Mycelium and spore suspension added to pots	Pot cultures in greenhouse (22–30 °C without additional light); field (Mid Europe)	Sand—1.7 (shoot and root fw)^a^	Serfling et al. (2007)
				Soil—1.4 and 1 (shoot and root fw)^a^	
				1.1 (straw yield on poor soil)^a^	
*Solanum lycopersicum*	4-week-old plants	Root dipping	Pot cultures in green house (22/19 °C; no additional light)	Nutrient solution—1.1 (shoot fw)	Fakhro et al. (2010)
				Sand—1.3 (shoot fw)	
				Commercial substrate—1.1 (shoot fw)	
*Chlorophytum* sp.	Micropropagated plantlets	Mycelium and spores mixed with soil	Polythene bags (greenhouse, 27 °C; 13 h light) → field (North India)	Greenhouse—1.3 (shoot and root dw)	Gosal et al. (2010)
				Field—1.1 (shoot dw)	
*Cicer arietinum*	Seeds	Seed coating with mycelium and spore suspension	Pots with soil (phytotron; 22–26 °C; 16 h light)	No effect	Meena et al. (2010)
*Cicer arietinum*	Seeds	Mycelium and spores mixed with soil	Pots with soil (greenhouse)	1.4 (total dry weight)	Nautiyal et al. (2010)
*Phaseolus aureus*	Seeds	Mycelium and spores mixed with soil	Greenhouse (24–31 °C, 11 h light)	1.4 (total dw 20 dai)	Ray and Valsalakumar (2010)
				0.6 (total dw 60 dai)	
*Brassica campestris*	5-day-old seedlings	Agar plug	15 days on MM medium, 1/2 strength, 12 h light; transfer to soil for 15 days	1.4 (root fw)	Sun et al. (2010)
				1.5 (shoot fw)	
*Piper nigrum*	Tissue cultured plants	Mycelium and spores mixed with sand	60 days in pot cultures	Effects on leave number (1.2) and fw (1.1), but not on dw	Anith et al. (2011)
*Glycine max*	Seeds	Soil cultures of the fungus	Not reported	1.1 (height) with cultivar JS-335	Rathod et al. (2011)
				no effect with cultivar TAMS-21	
*Foeniculum vulgare*	Seedlings	Mycelium and spores added to seedlings in pots (sand/peat/perlite)	15 days in greenhouse (25/18 °C, 16 h light)	1.3 (root fw)^a^	Dolatabadi et al. (2011a)
				1.2 (shoot fw)^a^	
*Thymus vulgaris*	Non-rooted cuttings	Agar plug	60 days on agar (25 °C)	3.5 (root fw); 3.7 (shoot fw)	Dolatabadi et al. (2011b)
			30 days on agar		
			120 days in substrate (sand/peat/perlite; 24/18 °C, 16 h light)	2 (root fw); 1.4 (shoot fw)	
*Vigna mungo*	Seeds	Seed coating	Greenhouse	2.7 (shoot dw)	Kumar et al. (2012a)
			Field	2.5 (shoot dw)	
*Fragaria* x *ananassa*	Micropropagated plantlets	Mycelium and spores added to seedlings in pots (vermiculite/peat/solirite)	(Phytotron; 28 °C; 16 h light)	1.3 (plant fw)	Husaini et al. (2012)
*Centella asiatica*	Rooted plantlets	Mycelium + spore	Liquid MS + PDB in glass bottles (23 °C; 16 h light)	1.4 (whole plants fw)	Satheesan et al. (2012)
				1.75 (whole plants dw)	

Repetitive experiments with the same plant species are not mentioned, except results were very different

^a^Approximate values deduced from graphs

Interestingly, root growth promotion can be achieved even in the absence of colonisation (Drüge et al. 2007). Therefore, it was not surprising to find *P*. *indica* producing the auxin indole-acetic acid (Sirrenberg et al. 2007). Although expression of auxin-regulated genes in *Arabidopsis* was not affected by the endophyte (Vadassery et al. 2008), such genes were induced in barley (Schäfer et al. 2009) and in Chinese cabbage (Lee et al. 2011), and their induction was causative for the strong growth-promoting effect. The inducer was, however, not the indole-acetic acid itself, but an unknown component in the exudates of the fungal hyphae (Lee et al. 2011). Ethylene typically inhibits plant growth, and some rhizobacteria produce enzymes that degrade ethylene (Hayat et al. 2010). In fact, *P*. *indica* seems to inhibit ethylene signalling, which could contribute to plant growth promotion (Barazani et al. 2007). The finding that although barley genes involved in ethylene synthesis are induced, ethylene-responsive genes repressed in *P*. *indica*-colonised roots further support this suggested role of the phytohormone (Schäfer et al. 2009). A more complex picture was drawn in *Arabidopsis*, where mutations in ethylene signal transduction components resulted in increased root colonisation and abolished growth promotion or even caused growth repression (Camehl et al. 2010). Hence, moderate interference with ethylene signalling may allow a certain degree of colonisation by releasing the inhibiting effect of the phytohormone. A total knock out, however, results in uncontrolled spread of *P*. *indica* and pathogenic behaviour. Additional phytohormones synthesised or manipulated by the root endophyte include cytokinins (Vadassery et al. 2008), gibberellins, abscisic acid and brassinosteroids (Schäfer et al. 2009). In response to colonisation, the abscisic acid pathway was proposed to enhance plant growth via calcium (Vadassery et al. 2009a), phosphoinositide and particular protein kinases (Camehl et al. 2011). In summary, nearly the whole orchestra of phytohormones and phytohormone signalling networks seems to be involved in generating compatible interactions between the fungus and host, which lead to increased early root growth promotion and finally to greater biomass.

## Plant nutrition

Rhizosphere microorganisms are able to support plant nutrition by two general mechanisms. They convert unavailable resources to plant available compounds, e.g. by nitrogen fixation or phosphate solubilisation, or they support their transport towards or even inside the plant root, both leading to increased mineral nutrient uptake by the plant (Hayat et al. 2010). In tobacco, barley and green gram, colonisation did not increase P or N content of plants although plant growth was promoted (Barazani et al. 2005; Achatz et al. 2010; Ray and Valsalakumar 2010). Chickpea and black lentil plants, however, showed higher N, P and K content (Nautiyal et al. 2010; Kumar et al. 2012a), and sugar cane plants could overcome Fe and Cu deficiencies if inoculated with the endophyte (Gosal et al. 2011). In *Arabidopsis*, uptake of radio-labelled P was strongly enhanced in the presence of the fungus (Shahollari et al. 2005), and such uptake was abolished in maize by down-regulation of one of the fungal phosphate transporters (Yadav et al. 2010). Similarly, a nitrate reductase shows enhanced expression in *P*. *indica*-colonised roots which could indicate that the fungus also supports N nutrition of plants (Sherameti et al. 2005).

At the moment, it appears as chicken-and-egg question: does *P*. *indica*-enhanced root development result in increased mineral nutrient uptake, or is more direct support of plant nutrition primarily responsible for the plant growth-promoting effects of colonisation? More targeted research is necessary to know which combinations of physical and chemical soil properties allow the fungus to contribute to plant nutrition and which of the two mechanisms mentioned above are involved. Especially important will be the analysis of conditions where the application of mineral fertilisers is reduced and exchanged with different types of organic matter that could serve as a resource for the saprophytic capabilities of *P*. *indica*. At least and in contrast to arbuscular mycorrhizal fungi, *P*. *indica* colonise plant roots independent of phosphate availability (Varma et al. 1999). This is a clear advantage for agricultural applications because the fungus can be applied even in anthropogenic ecosystems with high concentrations of phosphate where it also exerts plant growth-promoting effects (Achatz et al. 2010).

The need for more research also applies to the question of whether there is a reciprocal transfer of carbohydrates, and more specifically, whether the fungus establishes an additional carbohydrate sink for the plant. The induced expression of a starch-degrading enzyme in the roots (Sherameti et al. 2005) could be a hint, but enhanced degradation of starch might be also necessary for the accelerated development of the root and so expression may merely be regulated by metabolic needs rather than induced by the fungus. Such an additional sink could lead to carbohydrate starvation, but this could be balanced by higher CO_2_ assimilation rates in *P*. *indica*-colonised barley plants at low light intensities compared to the corresponding controls (Achatz et al. 2010). This goes along with an analysis of chlorophyll fluorescence showing *P*. *indica*-increased photosynthetic performance of maize plants (Rai et al. 2008).

## Abiotic and biotic stresses

The impact of biological agent application, including *P*. *indica*, on abiotic stress tolerance is the subject of numerous reports. Initial studies have explored how plants respond to the combination of stress factors and endophyte inoculation. If the positive impact of the endophyte is higher under stress conditions, then it can be concluded that the fungus confers stress tolerance on the plant. If the impact of the fungus is similar under stress and non-stress conditions, then it exerts its positive influence irrespective of the cultivation conditions. Decreased growth promotion by the endophyte under stress conditions implies that the plant–fungus interaction suffers in adverse conditions.

‘Positive’ interaction of abiotic stress and *P*. *indica* colonisation has been only shown for *Triticum aestivum* where the plant growth-promoting effect increased with rising salt concentrations (Zarea et al. 2012). In a different type of experiments, where factor interaction was not directly analysed, pre-inoculation with the endophyte relieved *Arabidopsis* seedlings from drought stress (Sherameti et al. 2008). Such seedlings continue to grow after water removal, but the development of non-inoculated controls is arrested. A similar phenomenon was observed in Chinese cabbage (Sun et al. 2010) and strawberry (Husaini et al. 2012). These observations cannot be simply explained by growth promotion.

In order to adapt to osmotic stress, plant tissues, e.g. of halophytes, accumulate organic solutes such as the amino acid proline (Moore 1975). Interestingly, *P*. *indica*-colonised plants have higher concentrations of proline than corresponding controls, and this could partially explain their increased tolerance to osmotic stress (Zarea et al. 2012). Another response to abiotic stress is the accumulation of reactive oxygen species (ROS) and the synthesis of corresponding antioxidants (Foyer and Shigeoka 2011), and it has been proposed that endophyte-conferred abiotic stress tolerance relies on an enhancement of these reactions (White and Torres 2010; Hamilton et al. 2012). Searching for the mechanisms of *P*. *indica*-induced stress tolerance also revealed increased conversion of dehydroascorbate to ascorbate and higher levels of glutathione, the two main antioxidants (Waller et al. 2005). Further analyses showed a significant interaction of the factors ‘*P*. *indica*’ and ‘salt’ on the expression of a number of enzymes involved in ROS metabolism in barley (Baltruschat et al. 2008). Such an enhanced expression was accompanied in Chinese cabbage by a clear reduction in malondialdehyde content, an indicator of unsaturated lipid degradation by ROS (Sun et al. 2010). Also the expression of a number of genes putatively involved in stress response is induced by drought to higher levels in plants colonised by *P*. *indica*. Interestingly, down-regulation of two genes encoding enzymes for ascorbate synthesis in *Arabidopsis* resulted in much greater colonisation by the endophyte and disappearance of its plant growth-promoting effect (Vadassery et al. 2009b).

Biotic stress protection by *P*. *indica* was first shown in barley roots against *Fusarium culmorum* and in shoots against *Blumeria graminis* (Waller et al. 2005). Since similar results have been obtained in plants from other families (Table 2), *P*. *indica* may protect a wide variety of plants against fungal pathogens. Root pathogens might be directly inhibited by antagonistic activities of the endophyte. Such growth inhibition of fungi by *P*. *indica* could not be observed for *F*. *culmorum* (Waller et al. 2005) or for *Pseudocercosporella herpotrichoides* (Serfling et al. 2007), but to a low extent for *Fusarium oxysporum* (Dolatabadi et al. 2012). At least for leaf pathogens, it is clear that *P*. *indica* root colonisation systemically induces resistance. The production of ROS and the synthesis of antioxidants seem to also play a role similar to the aforementioned abiotic stress protection, at least in the monocots barley, wheat and maize (Waller et al. 2005; Serfling et al. 2007; Kumar et al. 2009). Analysis of a number of *Arabidopsis* mutants showed that jasmonate signalling is important for *P*. *indica*-induced resistance (Stein et al. 2008). In root endophyte-colonised barley plants, a subset of defence-related genes is earlier and more strongly induced by leaf pathogens than in control plants (Molitor et al. 2011). Hence, the mechanisms of *P*. *indica*-induced resistance seem to be similar to the well-characterised induced systemic resistance described for plant growth-promoting rhizobacteria-colonised plants (van Wees et al. 2008). In contrast to fungal pathogens, colonisation of plants by the endophyte leads to a higher susceptibility to insect attack (Barazani et al. 2005) and results in increased viral spread at low light intensities in tomato (Fakhro et al. 2010).

**Table 2:** Protection against pathogens by *Piriformospora indica*

Plant	Pathogen	Evidence	Reference
*Hordeum vulgare*	*Fusarium culmorum*	Effect on shoot fresh weight^a^	Waller et al. (2005)
*Hordeum vulgare*	*Blumeria graminis*	Disease symptoms	
*Hordeum vulgare*	*Fusarium graminearum*	Pathogen spread	Deshmukh and Kogel (2007)
*Triticum aestivum*	*Pseudocercosporella herpotrichoides*	Disease symptoms	Serfling et al. (2007)
*Triticum aestivum*	*Blumeria graminis*	Disease symptoms	
*Arabidopsis thaliana*	*Golovinomyces orontii*	Disease symptoms	Stein et al. (2008)
*Zea mays*	*Fusarium verticillioides*	Effect on shoot dry weight^a^	Kumar et al. (2009)
*Solanum lycopersicum*	*Verticillium dahliae*	Disease symptoms	Fakhro et al. (2010)
*Solanum lycopersicum*	*Fusarium oxysporum*	Effect on dry root weight^a^	Sarma et al. (2011)
*Lens culinaris*	*Fusarium oxysporum*	Disease symptoms	Dolatabadi et al. (2012)

^a^Differences between *P*. *indica*-inoculated plants and controls were much higher in pathogen-inoculated plants

## Yield and product quality

In addition to positive effects on plant development, resistance and tolerance, *P*. *indica* colonisation also can improve crop plant yield, due to increased vegetative tissue yield, greater number of inflorescences and flowers (Rai et al. 2001; Dolatabadi et al. 2011a) or greater seed weight (Rai et al. 2001; Peskan-Berghofer et al. 2004; Barazani et al. 2005). For example, barley yield can be increased by 10 % depending on the cultivar, due to a higher number of ears (Waller et al. 2005), while in green gram, the number of pods per plant as well as the number of seeds per pod was higher (Ray and Valsalakumar 2010). In tomato, results depended on the harvest date. At early time points, twice as many fruits were harvested from colonised plants (Fakhro et al. 2010; Sarma et al. 2011), but over time non-colonised plants caught up and the same number of fruits was harvested (Andrade-Linares et al. 2012). In summary, plants with one, fixed-date of harvest seem to achieve higher yields, but in a plant like tomato with a long harvest period, the overall yield does not differ between *P*. *indica*-colonised plants and controls.

In addition to yield quantity, yield quality is also an important parameter. Chemical analyses showed increased concentrations of various compounds in *P*. *indica*-colonised plants such as the antifungal spilanthol in *Spilanthes calva* (Rai et al. 2004), pharmaceutically important substances such as podophyllotoxins from *Linum album* (Baldi et al. 2010), saponin from *Chlorophytum* sp. (Gosal et al. 2010) or asiaticoside from *Centella asiatica* (Satheesan et al. 2012) and essential oils in *Foeniculum vulgare* and *Thymus vulgaris* (Dolatabadi et al. 2011a, b). Moreover, as human health-promoting compounds such as antioxidants are shown to be increased in colonised plants (see above), *P*. *indica* may generally increase the quality of vegetables, fruits and seeds being used as food.

Micropropagated plantlets are another product that can benefit from the application of *P*. *indica*. Inoculation of such plantlets significantly increases the survival rate of tobacco, *Chlorophytum* species, sugar cane and strawberry (Varma et al. 1999; Sahay and Varma 1999; Mathur et al. 2008; Gosal et al. 2010, 2011; Husaini et al. 2012). This increased survival rate is probably due to both the promotion of root development and the increase in tolerance to abiotic stress.

## Comparison, combination and interaction with other microorganisms

As *P*. *indica* belongs to the *Sebacinales*, a group of endophytic fungi distributed worldwide (Weiß et al. 2011), it can be assumed that close relatives show similar effects on plant performance. Closely related to *P*. *indica* is the orchid mycorrhiza-forming species *Sebacina vermifera* (Weiß et al. 2004), and indeed *S*. *vermifera* isolates (deposited at the National Institute of Agrobiological Sciences, Tsukuba, Japan; culture collection numbers are described in Deshmukh et al. 2006) show similar effects on plant growth (Barazani et al. 2005, 2007; Ghimire et al. 2009; Baldi et al. 2010; Dolatabadi et al. 2011a, b), disease resistance (Deshmukh et al. 2006; Dolatabadi et al. 2012), drought tolerance (Ghimire and Craven 2011; Husaini et al. 2012) and chemical composition (Baldi et al. 2010; Dolatabadi et al. 2011a, b). Interestingly, a related multinucleate *Rhizoctonia* strain was isolated from an AM fungal spore (Williams 1985), as was *P*. *indica* (Verma et al. 1998). This strain was described as a new species, *Piriformospora williamsii* (Sharma and Kogel 2009; Basiewicz et al. 2012), but its impact on plant performance has not yet been analysed.

*P*. *indica* has also been compared and combined with other beneficial microorganisms. In direct comparison with AM fungi, *P*. *indica* did not increase P and N content of barley plants (Achatz et al. 2010) and showed less plant growth promotion in green gram (Ray and Valsalakumar 2010), but it increased the survival of micropropagated plants to a greater extent (Mathur et al. 2008). The mycoparasite *Trichoderma harzianum* inhibits *P*. *indica* growth in vitro and root colonisation, but inoculation of pepper plants with *P*. *indica* and subsequently with *T*. *harzianum* resulted in higher plant dry weights compared to single inoculations (Anith et al. 2011). Different combinations of *Trichoderma* species with *P*. *indica* and *S*. *vermifera* were tested for their effects on protection of lentil against *Fusarium* wilt, and the best effects were achieved by combining the two *Sebacinales* with *T*. *harzianum* (Dolatabadi et al. 2012). *P*. *indica* was also compared with various plant growth-promoting rhizobacteria (fluorescent pseudomonads, *Azospirillum* sp.), and they showed similar effects on growth promotion, yield, salt tolerance and disease resistance (Sarma et al. 2011; Kumar et al. 2012a; Zarea et al. 2012), while *P*. *indica* was superior to the bacteria in supporting the establishment of micropropagated plantlets (Gosal et al. 2010). However, again the strongest effects resulted from combining biological agents (Meena et al. 2010; Gosal et al. 2010; Sarma et al. 2011; Kumar et al. 2012a).

## Inoculum production and commercial application

In contrast to the obligate biotrophic arbuscular mycorrhizal fungi, *P*. *indica* can also be propagated in axenic cultures as a saprophyte and grows on numerous different natural and artificial cultivation media (Verma et al. 1998). However, the choice of substrate for inoculum production influences the impact on the plant (Andrade-Linares et al. 2012). The choice of N source, for example, is critical because propagation on a substrate containing only ammonium results in a strong negative effect on the plant after inoculation (Kaldorf et al. 2005).

In current experimental conditions, the fungus is typically applied as a mixture of hyphae and spores (Table 1). However, to produce inocula which can be commercially applied, it is necessary to obtain a larger quantity of spores. This can be achieved by optimisation of substrate composition and environmental conditions (Kumar et al. 2011) and by the application of certain nanomaterials (Suman et al. 2010). Additionally, for distribution, the inoculum must be combined with a carrier, and two such carriers have already been tested (Sarma et al. 2011). Other important parameters include the amount of inoculum being applied, the time point of inoculation (Fakhro et al. 2010) and the choice of soil or substrate for plant cultivation (Serfling et al. 2007; Fakhro et al. 2010).

Further, to place *P*. *indica* on the market, it must be registered as an inoculum. Regulations for registration vary between countries, but one bottleneck concerning safety might be that sometimes negative effects on plant growth can be observed. This is probably based on the mode of colonisation, which includes a dependency on programmed cell death (Deshmukh et al. 2006). Another concern is that the fungus was isolated in India, and at present, only this one isolate of *P*. *indica* exists. Because *S*. *vermifera* is distributed worldwide and shows similar characteristics to *P*. *indica* (see above), it will be useful to obtain more *S*. *vermifera* isolates from different regions and to analyse their impact on the plant and for their mode of colonisation. The fact that the hyphae of *P*. *indica* and related *Sebacinales* contain bacteria, which promote plant growth and disease resistance and which can at least partially be cultivated (Sharma et al. 2008), opens up the possibility of using such bacteria as inoculum. Another alternative would be the application of culture filtrate, since such filtrate can also promote plant growth and development (Varma et al. 1999; Ghimire et al. 2009; Vadassery et al. 2009a; Bagde et al. 2011; Kumar et al. 2012b) and influences the synthesis of particular valuable compounds (Balsi et al. 2010; Bagde et al. 2011; Kumar et al. 2012b).

## Outlook

The interaction of *P*. *indica* with plant roots has been intensively studied, and genome sequence and transformation systems are available. However, in order to use the root endophyte in agricultural practice, a product for commercial use must be established and registered. For this purpose, future research concerning applications should concentrate on the following points:

- Evaluation of alternatives to *P*. *indica*, including related fungal isolates, endophytic bacteria and culture filtrate
- Inoculum production conditions
- Inoculum formulation and stability
- Persistence of the fungus in the environment

After which, it will be possible to specify scopes for application, which theoretically could be manifold as the present review has shown, and to define the conditions which support the beneficial effects. Finally, it will be necessary to calculate ecological and economic costs and benefits to guide *P*. *indica* and related products to successful agricultural application.
